# Fetal head size and effect of manual perineal protection

**DOI:** 10.1371/journal.pone.0189842

**Published:** 2017-12-29

**Authors:** Magdalena Jansova, Vladimir Kalis, Zdenek Rusavy, Sari Räisänen, Libor Lobovsky, Katariina Laine

**Affiliations:** 1 New Technologies—Research Centre, University of West Bohemia, Pilsen, Czech Republic; 2 Biomedical Centre, Faculty of Medicine in Pilsen, Charles University, Pilsen, Czech Republic; 3 Department of Obstetrics and Gynecology, University Hospital, Pilsen, Czech Republic; 4 University of Helsinki, Helsinki, Finland; 5 National Library of Finland, Helsinki, Finland; 6 NTIS—New Technologies for Information Society, Faculty of Applied Sciences, University of West Bohemia, Pilsen, Czech Republic; 7 Department of Obstetrics, Oslo University Hospital, Oslo, Norway; Monash University, AUSTRALIA

## Abstract

**Objective:**

The aim of this study was to evaluate whether a previously identified modification of Viennese method of perineal protection remains most effective for reduction of perineal tension in cases with substantially smaller or larger fetal heads.

**Methods:**

A previously designed finite element model was used to compare perineal tension of different modifications of the Viennese method of perineal protection to "hands-off" technique for three different sizes of the fetal head. Quantity and extent of tension throughout the perineal body during vaginal delivery at the time when the suboccipito-bregmatic circumference passes between the fourchette and the lower margin of the pubis was determined.

**Results:**

The order of effectiveness of different modifications of manual perineal protection was similar for all three sizes of fetal head. The reduction of perineal tension was most significant in delivery simulations with larger heads. The final position of fingers 2cm anteriorly from the fourchette (y = +2) consistently remains most effective in reducing the tension. The extent of finger movement along the anterior-posterior (y-axis) contributes to the effectiveness of manual perineal protection.

**Conclusion:**

Appropriately performed Viennese manual perineal protection seems to reduce the perineal tension regardless of the fetal head size, and thus the method seems to be applicable to reduce risk of perineal trauma for all parturients.

## Introduction

Obstetric anal sphincter injury (OASI) is a severe complication that may occur in otherwise uncomplicated vaginal delivery. Up to 60% of women suffer from anal incontinence after OASI [1–3]. Increased occurrence of perineal pain and discomfort, and also sexual disorders were reported after OASI compared to controls [4,5]. A steep increase in the incidence of OASI has been observed in many countries recently [6–9]. To reverse this unfavorable trend, modifiable risk factors have been extensively evaluated [10–12].

Methods of manual perineal protection (MPP) have not been consistently defined and several different methods are used in clinical practice. This might explain why previous randomized controlled trials [13,14], considered as the best scientific evidence, have not found perineal manipulation procedures to be beneficial, whereas observational studies utilizing properly defined MPP training for all staff and MPP for all women advocate the benefit of the method [11,12,15–17].

Historically, manual perineal protection was suggested as an aid in decreasing the rate and degree of perineal injury [18,19]. However, only six of the 42 described modifications of MPP used thumb and index finger and only two of them reported active coordination between the two fingers [19]. One of these is the Viennese modification (VMPP) where the tips of the thumb and index finger are applied on the skin alongside the fourchette and vaginal orifice and these fingers are pressed against the perineum and a region of parietal eminences of the fetal head and moved towards each other while staying in a contact with the parturient's perineal skin. The main principle of this perineal protection modification is to disperse the highest perineal tension over a wider surface area [20,21], i.e. the reduction of transverse perineal tension by application of accoucheur's thumb and index finger laterally of the vaginal opening.

The pitfall of clinical obstetrics is that MPP cannot be evaluated separately as it is always performed together with other obstetric interventions. Therefore, one single modification of MPP should be clearly defined before its appropriate clinical evaluation. A simulation of vaginal delivery using a novel biomechanical model showed a significant reduction in perineal tension when an appropriate modification of VMPP was applied [20].

The aim of the present study was to evaluate whether this simulated VMPP remains effective method for reduction of perineal tension in cases with different sizes of fetal head.

## Materials and methods

This study was a part of a larger project: Perineal Trauma Prevention, Evaluation, Education and Recognition Study Group: Perineal Protection Program incorporating the Principles of Physics (PEERS 5P‘s).

### Virtual birthing model: Its characteristics and modelling technique

In order to predict the behavior of tissue under load, it is possible to compose a virtual (computational) birthing model. Such analysis of the behavior of tissues under load has become possible in the recent development in computing. A quasi-incompressible transversely isotropic hyperelastic Mooney-Rivlin material model for the soft tissue, as described in a previous study [20], was used for the simulations. The three-dimensional mesh was composed of 162,000 tetrahedral elements of the mean edge size of 2mm. The model geometry and the computational mesh were generated using a HyperMesh software package (Altair, Troy, MI, USA). Virtual-Performance Solution software was used for the simulations (Esi Group, Paris, France) [22].

**Fig 1:**
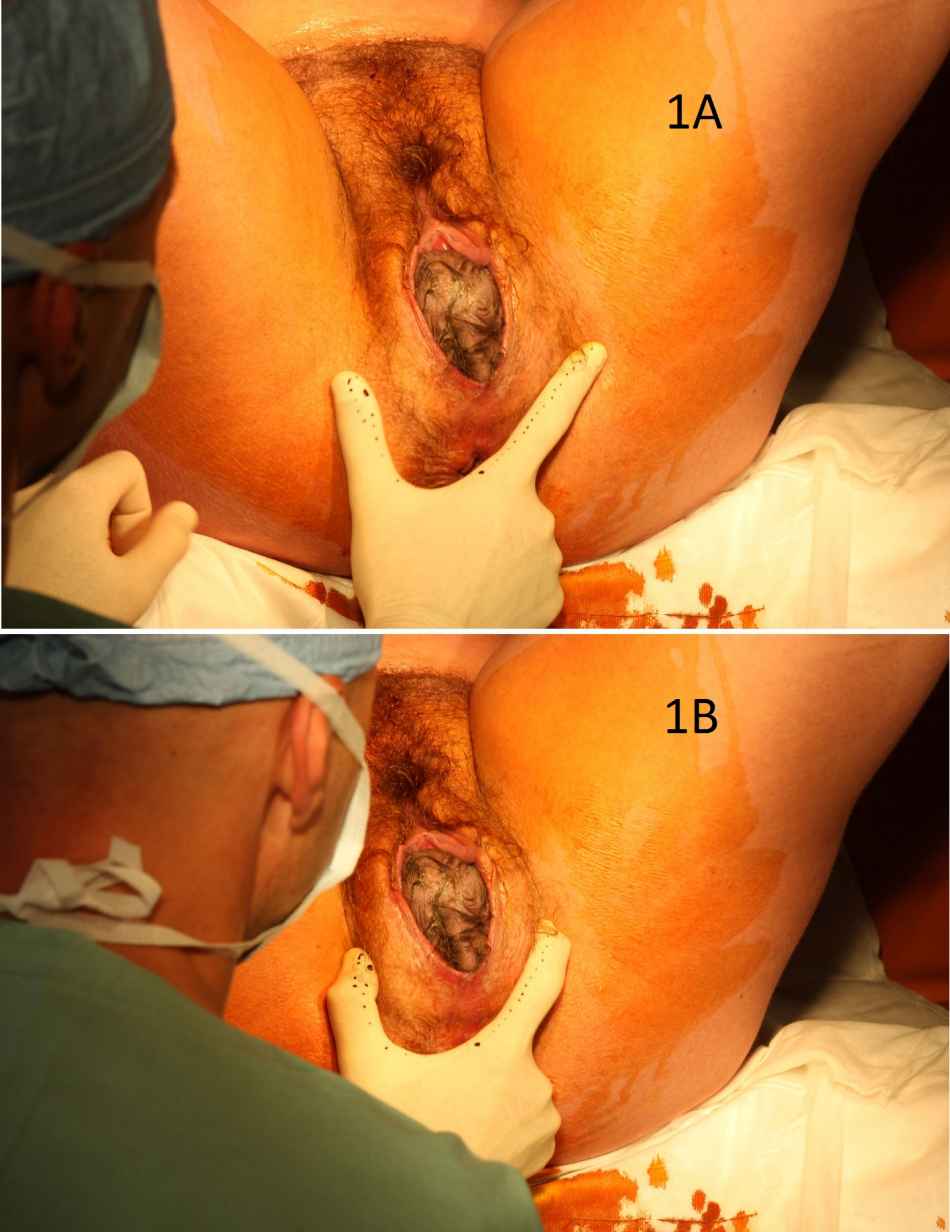
The most effective modification of VMPP calculated from numerical model during the expulsion of an average-sized fetal head. NB The initial position of fingers is 12 cm apart and 2 cm anteriorly from the posterior fourchette (Fig 1A). The fingers, still in contact with the perineal skin, are subsequently moved from each side 1 cm towards the midline. No movement in an antero-posterior dimension is performed (Fig 1B).

In our previous PEERS 5P‘s studies with the virtual birthing model, experimental measurements revealed that the placement of fingers on the perineal skin together with their coordinated movement plays an important role in the reduction of perineal tension and that the extent of this reduction varies considerably between different modifications [23]. In the most effective modification of VMPP, the initial position of fingers was 12 cm apart and 2 cm anteriorly from the posterior fourchette (Fig 1A) with a bilateral 1 cm movement towards the midline and no movement in an antero-posterior direction (Fig 1B). During the testing, fingers still in contact with the perineal skin are subsequently moved from each side 1 cm towards the midline. No movement in an antero-posterior dimension is performed (Fig 1B) [23].

**Fig 2:**
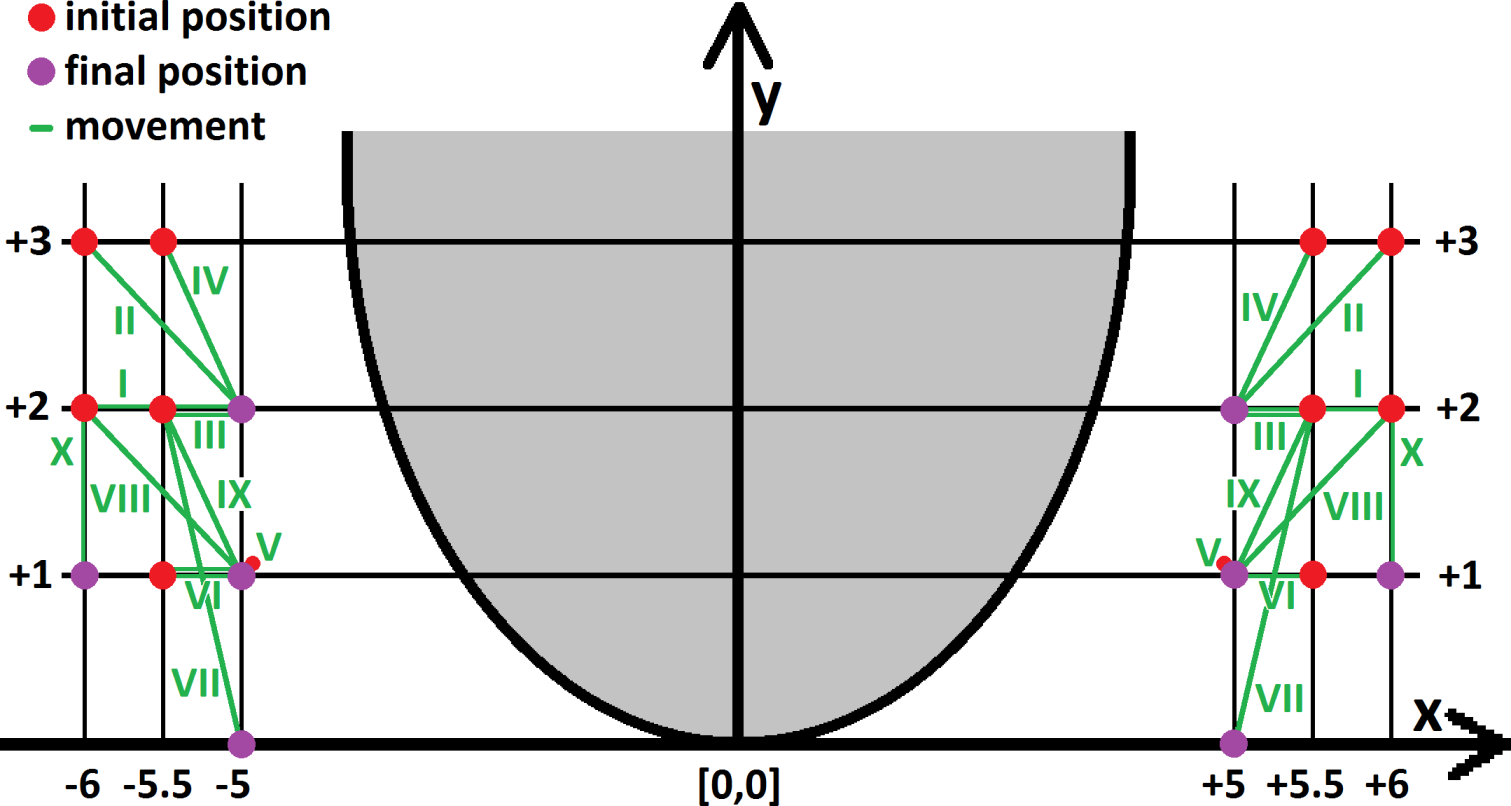
Initial placement of fingers and vectors of subsequent coordinated movements in evaluated modifications of MPP.

The model enables a depiction of the stretching and movement of the perineal tissue around the fetal head during a simulation of vaginal delivery. The fingers were applied when the antero-posterior diameter of the vaginal introitus was 7 cm and the transverse diameter was 5.3 cm (Fig 2). The referential points for defining the exact location of the fingers on the perineum were the foci) of their imprints. The trajectory of the passage of the fetal head through the birth canal followed the ideal Curve of Carus, i.e. during the expulsion the head pivoted as closely as possible around the lower margin of the pubic symphysis.

Determination of the exact placement location of the fingers on the perineum [x,y] was made using a coordinate system at an axial plane of the perineum and fetal head with its origin at the posterior fourchette [0,0]. The coordinates, x- and y-axes, were defined as horizontal and vertical lines crossing the referential point [0,0] (Fig 2). The coordinated movement between the fingers was performed along these axes [Δx,Δy] [23].

### Virtual fetal head characteristics and simulations of vaginal delivery

For the present study the finite element model [20] designed on the basis of data from previous studies [23,24–32] was used to analyze the perineal tension for three different sizes of fetal head during vaginal delivery. The molded average sized fetal head was defined by molded biparietal diameter (mBPD) and molded suboccipito-bregmatic diameter (mSOBD): mBPD = 91mm, mSOBD = 94mm, for the smaller head the dimensions were: mBPD = 87mm, mSOBD = 90mm, while the larger head was defined as mBPD = 95mm, mSOBD = 98mm [33].

We used ten VMPP modifications regarding finger placement and coordinated movement that were previously demonstrated to be the most effective in the reduction of peak perineal tension in case of average sized fetal head (Fig 2, Table 1) [23]. The observed perineal tension for each modification of VMPP was compared to the "hands-off"-technique.

**Table 1:** The placement of the thumb and index finger in the ten performed simulations, their subsequent coordination and final position.

Simulation	Initial placement (x-axis) [cm]	Initial placement (y-axis) [cm]	Transverse movement on each side [Δx] [cm]	Antero-posterior movement [Δy] [cm]	Final placement (x-axis) [cm]	Final placement (y-axis) [cm]
**I**	12 (-6, +6)	+2	1	0	10 (-5.0, +5.0)	+2
**II**	12 (-6, +6)	+3	1	1	10 (-5.0, +5.0)	+2
**III**	11 (-5.5, +5.5)	+2	0.5	0	10 (-5.0, +5.0)	+2
**IV**	11 (-5.5, +5.5)	+3	0.5	1	10 (-5.0, +5.0)	+2
**V**	10 (-5.0, +5.0)	+1	0	0	10 (-5.0, +5.0)	+1
**VI**	11 (-5.5, +5.5)	+1	0.5	0	10 (-5.0, +5.0)	+1
**VII**	11 (-5.5, +5.5)	+2	0.5	2	10 (-5.0, +5.0)	0
**VIII**	12 (-6, +6)	+2	1	1	10 (-5.0, +5.0)	+1
**IX**	11 (-5.5, +5.5)	+2	0.5	1	10 (-5.0, +5.0)	+1
**X**	12 (-6, +6)	+2	0	1	12 (-6, +6)	+1

The ten most effective modifications of VMPP calculated for the molded normal head [23] (Table 1) were applied to a delivery simulation of a smaller and a larger head. The simulations were labeled by Roman numerals (I-X) and sorted according to their effectiveness at reduction of the peak perineal tension that was calculated for the average head size. Simulation I was the most effective and simulation X the least effective of this selected set of modifications. Simulation 0 was reserved for the "hands-off" technique.

The initial placements of the thumb and index-finger [x,y] were: 10–12 cm apart on the x-axis (lateral direction) and at 1–3 cm on the y-axis (anterior-posterior direction) [23] (Table 1 and red points on Fig 2).

The movement of each of the virtual fingers on the perineal skin [Δx,Δy] was 1 cm (simulations I, II, VIII), 0.5 cm (simulations III, IV, VI, VII, IX) or 0 cm (simulation V, X) towards the midline from each side (along the x-axis) and 2 cm (simulation VII), 1 cm (simulations II, IV, VIII—X) or 0 cm (simulations I, III, V, VI) posteriorly along the y-axis [23] (Table 1 and Fig 2).

### Outcome / measurements

**Fig 3:**
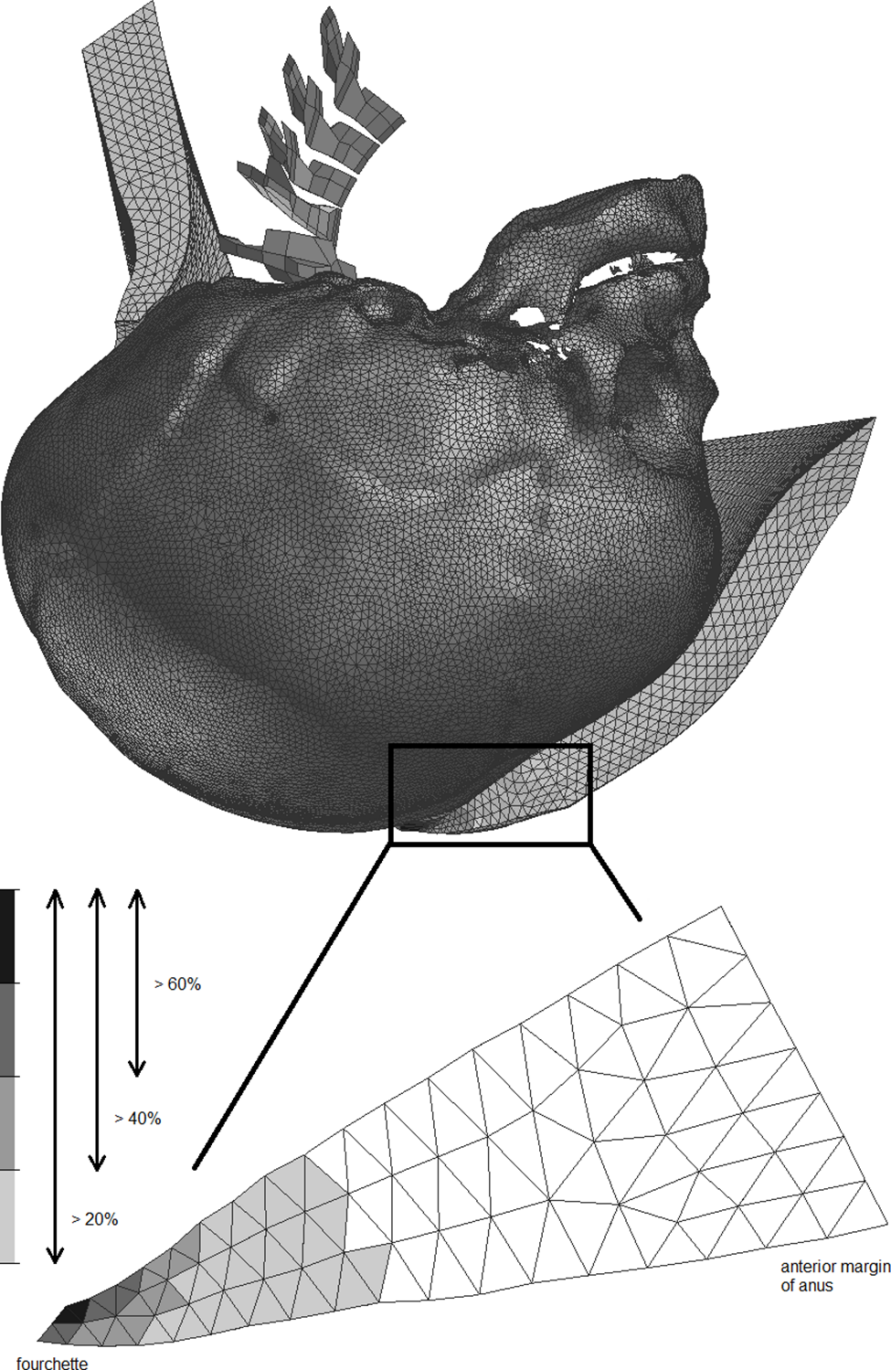
Mid-sagittal plane of the segment of the perineum during the "hands-off" simulation and stress distribution in the tissue at the moment of fetal head expulsion with areas where the tension exceeded 20%, 40% and 60% of the maximum tension (i.e. 100%).

The main outcome, perineal tension was measured during the video-simulation when the suboccipito-bregmatic circumference was passing between the fourchette and the lower edge of the pubic bone. A scale was used for digital visualization of the relative perineal tension where 100% corresponds to the maximum stress in the “hands-off”technique (Fig 3).

When the suboccipito-bregmatic circumference was passing between the lower margin of the pubic symphysis and the posterior fourchette during the simulated expulsion, the perineal tissue tensions at the fourchette were compared with respect to different modifications (including "hands-off") and different sizes of the molded fetal head. In addition, areas on the cross-section through the mid-sagittal plane, where the increment of perineal tension exceeded 20%, 40% and 60% of the maximum tension achieved during the "hands-off" simulation (Fig 3), were compared.

No statistical analysis was applicable due to the character of this study. As the model enables infinite number of simulations any difference in measurements would become statistically significant.

## Results

Modification I of VMPP remained consistently most effective in reducing the perineal tension regardless of the size of the fetal head. The second, third and fourth best modifications (II, III and IV, respectively), retained these positions in all measurements irrespective of the size of the fetal head (Tables 2 and 3) i.e. the order of effectiveness of the studied VMPP modifications was nearly identical for all three sizes of the fetal head (Tables 2 and 3).

**Table 2:** The order of effectiveness of VMPP modifications (in brackets), relative perineal tension at the fourchette during expulsion of small, normal and large fetal head. The relative perineal tissue tension provided in percentage with the pre-set maximum tension in the "hands-off" model at 100% and the pre-set tension at rest at 0%.

Simulation	Normal head (mBPD = 91mm)	Small head (mBPD = 87mm)	Large head (mBPD = 95mm)
	Perineal tension at fourchette at fetal head expulsion [%] (order of effectiveness)		
**0** **Hands off**	100.0 (11)	100.0 (9)	100.0 (11)
**I**	72.1 (1)	82.5 (1)	70.1 (1)
**II**	76.9 (2)	88.3 (3)	78.3 (4)
**III**	77.9 (3)	85.7 (2)	76.0 (3)
**IV**	81.1 (4)	89.0 (4)	70.1 (1)
**V**	85.3 (5)	90.9 (5)	82.0 (5)
**VI**	85.6 (6)	90.9 (5)	85.7 (7)
**VII**	86.9 (7)	108.5 (11)	95.4 (10)
**VIII**	88.4 (8)	93.5 (7)	82.5 (6)
**IX**	88.4 (8)	101.3 (10)	89.0 (9)
**X**	90.0 (10)	96.8 (8)	88.0 (8)

**Table 3:** The order of effectiveness of VMPP modifications (in brackets) according to the sizes of areas with aggregate proportionate tension for each simulation (in divisions of 20%, i.e. ≥20%, ≥40%, and ≥60% of maximum perineal tension during "hands-off") where the area of the whole segment of the perineum is 100% (Fig 3).

Simulation	Normal head (mBPD = 91mm)			Small head (mBPD = 87mm)			Large head (mBPD = 95mm)		
	Area of increment of perineal tension [%] (order of effectiveness)			Area of increment of perineal tension [%] (order of effectiveness)			Area of increment of perineal tension [%] (order of effectiveness)		
	>20%	>40%	>60%	>20%	>40%	>60%	>20%	>40%	>60%
**0** **Hands off**	20.4 (11)	3.5 (11)	1.8 (11)	26.2 (11)	5.2 (11)	1.9 (11)	18.2 (11)	3.0 (11)	1.5 (11)
**I**	8.9 (1)	1.8 (1)	0.3 (1)	12.8 (1)	3.2 (2)	0.3 (1)	8.0 (1)	1.7 (1)	0.3 (1)
**II**	9.5 (2)	2.4 (2)	0.3 (1)	13.0 (2)	3.2 (2)	1.2 (3)	8.5 (3)	2.0 (3)	0.3 (1)
**III**	12.8 (6)	2.9 (3)	0.3 (1)	14.2 (4)	3.3 (7)	1.2 (3)	8.7 (4)	2.2 (4)	0.3 (1)
**IV**	13.1 (8)	3.2 (8)	0.3 (1)	15.3 (6)	3.2 (2)	1.2 (3)	8.1 (2)	1.7 (1)	0.3 (1)
**V**	13.4 (9)	3.3 (9)	1.2 (10)	20.3 (10)	3.4 (8)	1.3 (8)	11.9 (10)	2.8 (5)	0.3 (1)
**VI**	12.8 (6)	3.1 (7)	1.1 (6)	15.3 (6)	3.2 (2)	1.2 (3)	11.8 (8)	2.9 (10)	1.1 (10)
**VII**	19.0 (10)	3.4 (10)	1.0 (5)	19.9 (9)	4.9 (10)	1.8 (9)	11.8 (8)	2.8 (5)	1.0 (7)
**VIII**	12.3 (3)	3.0 (4)	1.1 (6)	14.0 (3)	3.1 (1)	1.1 (2)	9.2 (5)	2.8 (5)	0.8 (6)
**IX**	12.5 (4)	3.0 (4)	1.1 (6)	19.2 (8)	3.6 (9)	1.8 (9)	11.0 (7)	2.8 (5)	1.0 (7)
**X**	12.6 (5)	3.0 (4)	1.1 (6)	15.1 (5)	3.2 (2)	1.2 (3)	10.9 (6)	2.8 (5)	1.0 (7)

The strongest effect of the presented modifications was during a delivery simulation with the larger head. Here, the most effective VMPP reduced the maximum perineal tension to below the values measured in case of the "hands-off" approach on a smaller fetal head (Table 4). The effectiveness of the best modification in simulated deliveries of a larger head was higher than all but two modifications in simulations with an average size of fetal head (Table 4).

**Table 4:** Direct comparison between perineal tensions of a variety of MPP simulations with respect to different sizes of the fetal head and between normal fetal head expulsion without any intervention. The maximum tension during the "hands-off" simulation with normal fetal head is the referrential tension, hence the proportion for this simulation is 1.00. The lower the number the higher the efficiency of the simulated intervention.

Simulation	Normal head (mBPD = 91mm)	Small head (mBPD = 87mm)	Large head (mBPD = 95mm)
**0** **Hands off**	1.00	0.81	1.14
**I**	0.72	0.67	0.80
**II**	0.77	0.72	0.90
**III**	0.78	0.70	0.87
**IV**	0.81	0.72	0.80
**V**	0.85	0.74	0.94
**VI**	0.86	0.74	0.98
**VII**	0.87	0.88	1.09
**VIII**	0.88	0.76	0.94
**IX**	0.88	0.82	1.02
**X**	0.90	0.78	1.01

The present study showed that the exact placement of the fingers play a major role in the reduction of maximum perineal tension. To perform an efficient MPP, the thumb and the index finger must be positioned sufficiently anteriorly and apart. The consistently most effective modification of VMPP for all studied sizes of the fetal head (simulation I) was when the fingertips are initially placed 12 cm apart (x = ±6) on the x-axis and 2 cm anteriorly from the posterior fourchette (y = +2) on the y-axis and both the index-finger and the thumb are subsequently moved 1 cm towards the midline along the x-axis (Δx = 1). The final position of fingers (x = ±5; y = +2) was identical for all four best modifications (simulations I-IV) regardless of the size of the fetal head (Tables 2, 3 and 4; Fig 2).

## Discussion

This computational study on a virtual model suggests that utilization of VMPP is beneficial regardless of the size of the fetal head. The VMPP modification established to be the most effective in reducing of perineal tension for the average sized head (simulation I) and remained also the most efficient for the delivery of substantially smaller or larger fetal head. This seems to be applicable for daily routine practice and supports the concept that MPP should be performed in all deliveries and not only be reserved for high risk deliveries with macrosomic fetuses. Regarding utilization of the methods in clinical practice, the four best modifications of VMPP (simulations I-IV) are very similar and easy to adopt for any individual, including those with shorter fingers or those with problems with precise quick identification of initial best position of fingertips. Thus, exact initial position of fingers and the extent of their movement plays an important role in the effectiveness of VMPP. One cm difference in distance of the fingers is responsible for up to 30% of the relative difference in perineal tension between modifications [23].

This study demonstrated that the reduction of perineal tension was most efficient in simulations with larger fetal head, i.e. during a delivery of a larger fetus. This more profound reduction is understandable based on two facts. Firstly, the larger the head the higher the tension on the perineum when delivering "hands-off". Secondly, if the accoucheur is able to make the movement with his/her fingers on the perineum when executing MPP to the same extent as in case of delivery of a normal head then the proportionate reduction of maximum tension must be more profound. Moreover, the proper performance of the best modification in case of a large fetal head is capable of achieving lower perineal tension than "hands-off" in case of a small fetal head. Consequently, MPP seems to be applicable for daily routine practice and supports the concept that MPP should be performed in all deliveries and not only be reserved for deliveries with macrosomic fetuses with higher risk of OASIS.

Recently, MPP has been somewhat neglected, possibly due to few studies that demonstrated the "hands-off" technique to be associated with comparable or lower perineal injury rate. These previous randomized controlled trials [13,14] suggesting that MPP is not beneficial have been repeatedly criticized for methodological inaccuracies, for poor control and lacking description of MPP [15–17,20–21,23]. An exact, detailed and reproducible definition of MPP has never been sufficiently provided. A search is underway for optimal interventions aimed at reduction and prevention of perineal injury associated with vaginal delivery. The recent theoretical [20,23] and clinical knowledge [11,12,15–17,34] have demonstrated that MPP was the major intervention used to successfully reverse the increasing trend in the rate of OASIS. In Norway, a large interventional project was started in the mid-2000s in several hospitals with the high OASIS rate where correct MPP and episiotomy techniques were applied for all the women regardless of the risk status. As a result the OASIS rates reduced at least by 50% in all the subgroups, such as women with operative deliveries and large babies [12, 15–17]. Generally, increased professional awareness and engagement in quality improvement via scientific approach, such as the STOMP project [35] may result in significant decrease of the numbers of severe perineal trauma and its consequences.

The advantage of the computational modeling compared to clinical studies is that the tension can be measured precisely at any moment during the simulation, which is not feasible in clinical practice. Another important advantage is that the process is repeatable and same results are obtained if no change in variables has been made. This allows the altering of only one or two variables at time, and also an analysis to be carried out separately from the other variables in order to quantitatively evaluate the individual and/or composite effect.

The computational modeling facilitates a detailed analysis of all steps performed during MPP, accuracy of timing of measurement and precise quantitative measurement of perineal tension during simulated vaginal delivery. It has been shown that from biomechanical principles the exact placement and coordinated movement of the fingers on the perineum when performing MPP is very important [20,23]. The results of this presented study confirmed that MPP, when done properly, is effective in reducing perineal tension regardless of the size of the fetal head. The results in our study support the findings of clinical studies where the risk of perineal injuries was reduced by re-introducing MPP as a routine intervention during vaginal delivery, and the entire staff was trained to use standardized MPP technique altogether with improved episiotomy technique and manual and verbal control of the fetal head expulsion [11,12,15–17].

The main limitations, such as possible inaccuracies inherent in the material and its parameters were discussed in our previous studies [20,23]. In accordance with the review of other studies [36], a hyperelastic material model was adopted. The disadvantage of this material is that it is not dependent on the loading history, i.e. this study evaluated the role of the dominant-posterior hand on the perineum, and not the effect of the other hand slowing the speed of the head passage through the perineal structures. The objective was to assess the relative effectiveness of several modifications of VMPP (accoucheur's dominant hand) for different sizes of the fetal head. As the models only differ in regards to fetal head size, this assessment was not affected by the selection of the material [23].

The biophysical properties of the perineum are given by its dimensions, structure and mechanical properties of its tissues. Due to the lack of information about the exact mechanical properties of specific perineal tissues, the perineal body is modeled as a homogenous structure, which is a simplification limiting the results. However, the adopted model is capable of predicting clinically observed results such as the most probable location of perineal tear in the region of the fourchette in mid-posterior perineum [21]. In reality the tear in this location may not only be caused by the highest values of perineal strain (observed in the fourchette) [21], but also by a different infrastructure of the perineal central tendon (a fusion of perineal muscles and perineal membrane) which could be more fragile than the lateral regions (perineal membrane and muscles separated as different entities). The universal distribution of mechanical properties can be considered a weakness of the model. However, during simulation the distribution of the tissue strain measured on the model corresponds to that observed in clinical experiments, which were performed on the perineum during the crowning of the fetal head in our previous study [21]. Therefore, in spite of not knowing the exact material properties and using a homogenous model, the model acts realistically and the results of the testing can be extrapolated in a general sense. The conclusion that a specific placement and coordinate movement of the fingers on the perineal surface decreases the peak tension in the midline of the posterior fourchette can thus be reached.

## Conclusions

Our study using a computational model shows a reduced perineal tension when the correct MPP technique is used. The most effective MPP with the fingertips initially placed 12 cm apart and 2 cm anteriorly from the posterior fourchette, and with a subsequent movement one cm on each side towards the midline without any anterio-posterior shift should be easily acquired and clinically implemented. We also conclude that the MPP is useful and beneficial for all parturients with different sizes of neonates. Our results facilitate to understand and may partly provide explanation of results of clinical studies where OASI incidence was reduced by educating the delivering staff to use MPP for all parturients [11,12,15–17].

Further studies ought to evaluate whether, and to what extent, the optimal placement and coordinated movement of the fingers during VMPP differ for a variety of uncommon or complicated deliveries (prolonged second stage, perineal edema, abnormal fetal head presentation, breech, etc.).
